# *In vitro* activities of aztreonam/nacubactam and cefepime/nacubactam against a global collection of molecularly characterized Enterobacterales isolates carrying carbapenemases: a global antimicrobial resistance surveillance study from 2021 to 2023

**DOI:** 10.1093/jacamr/dlag166

**Published:** 2026-08-12

**Authors:** Mark Estabrook, Eisuke Suzuki, Atsuyuki Shimizu, Hayato Okade

**Affiliations:** IHMA Inc., Schaumburg, IL 60173, USA; Meiji Seika Pharma Co., Ltd, 2-4-16, Kyobashi, Chuo-ku, Tokyo 104-8002, Japan; Meiji Seika Pharma Co., Ltd, 2-4-16, Kyobashi, Chuo-ku, Tokyo 104-8002, Japan; Meiji Seika Pharma Co., Ltd, 2-4-16, Kyobashi, Chuo-ku, Tokyo 104-8002, Japan

## Abstract

**Objectives:**

Nacubactam is a novel diazabicyclooctane β-lactamase inhibitor with activity against PBP2 that is in clinical development with aztreonam or cefepime. The *in vitro* activities of aztreonam/nacubactam, cefepime/nacubactam and comparator agents were determined against carbapenemase-producing Enterobacterales, collected globally from 2021 to 2023.

**Methods:**

Isolates were collected as part of a surveillance study from 24 countries in Asia, Europe and North America. Susceptibility testing was by CLSI broth microdilution. Meropenem-non-susceptible Enterobacterales isolates were sequenced by nanopore technology to identify resistance mechanisms. Of 12 613 isolates collected, 630 carbapenemase-producing isolates were detected.

**Results:**

All KPC-producing (158) and OXA-48-like–producing (142) isolates were inhibited by ≤4/4 mg/L of aztreonam/nacubactam or ≤8/8 mg/L cefepime/nacubactam. Aztreonam/nacubactam at ≤4/4 mg/L was active against 98.7% of the 307 NDM-producing isolates, more than aztreonam/avibactam (95.1% susceptible) or cefiderocol [85.7% susceptible (CLSI breakpoints)], while cefepime/nacubactam was active against 94.1% at ≤8/8 mg/L. Insertions in PBP3 were present in 39/47 (83%) of NDM-positive *Escherichia coli*. Aztreonam/nacubactam was active against 97.4% of these at ≤4/4 mg/L and cefepime/nacubactam was active against 100% at ≤8/8 mg/L, while aztreonam/avibactam and cefiderocol activities were relatively poor [69.2% aztreonam/avibactam-susceptible and 46.2% cefiderocol-susceptible (CLSI)].

**Conclusions:**

Aztreonam/nacubactam and cefepime/nacubactam demonstrated potent *in vitro* activity against Enterobacterales that carried serine-carbapenemases and MBLs, including those with chromosomal resistance mechanisms that compromise the current best-in-class β-lactam antibiotics for treating infections caused by MBL-positive organisms. The percentage of NDM-positive *E. coli* with insertions in PBP3 is alarming and warrants continued surveillance.

## Introduction

Enterobacterales are significant in urinary tract infections, hospital/ventilator-acquired bacterial pneumonia and intra-abdominal infections as well as other types of infections.^1,2^ The global spread of β-lactamases among Enterobacterales threatens the effectiveness of one of the most reliable classes of broad-spectrum antibiotics used to treat infections caused by these organisms.^3,4^ In recent years, the rapid global dissemination of carbapenem-resistant Enterobacterales (CRE), particularly those that carry MBLs, has been a primary concern.^3^ MBLs such as variants of New Delhi metallo-β-lactamase (NDM) hydrolyse all classes of clinically available β-lactam antibiotics except for aztreonam.^5^

Nacubactam is a diazabicyclooctane β-lactamase inhibitor under development with aztreonam or with cefepime for the treatment of infections due to CRE (Integral-1 trial, NCT05887908; Integral-2 trial, NCT05905055). Two global Phase 3 trials have been completed, and preparations for the regulatory submission are underway at the time of this writing. Nacubactam is described as a β-lactam enhancer (BLE) because it not only inhibits β-lactamases, potentially improving the activity of the co-administered β-lactam, but it also has antimicrobial activity through inhibition of PBP2.^6^ Nacubactam has inhibitory activity against Ambler class A, C and some class D β-lactamases, but not class B MBLs. Perhaps primarily due to its class A β-lactamase inhibitory activity, nacubactam has been shown to increase the activity of meropenem against KPC-producing Enterobacterales *in vitro*.^7^ It has also been shown to enhance the *in vitro* activity of aztreonam, cefepime and meropenem against MBL-producing Enterobacterales.^6^ This may be achieved through the inhibitory activity of nacubactam on PBP2, or in the case of aztreonam, protection of the monobactam from hydrolysis by additional class A ESBLs that are frequently co-carried with MBLs.^8^

Numerous β-lactam/β-lactamase inhibitor combinations have been developed for the treatment of infections caused by Enterobacterales that carry class A carbapenemases (KPC and select variants of GES, among the most clinically relevant). Such combinations include ceftazidime/avibactam, imipenem/relebactam and meropenem/vaborbactam, none of which display activity against organisms that carry MBLs. There are limited approved therapeutic options to treat infections caused by MBL-positive Enterobacterales at the time of this writing. Two options that frequently exhibit *in vitro* activity against MBL-positive Enterobacterales are cefiderocol and aztreonam/avibactam. Cefiderocol was approved by the US FDA in 2019^9^ and by the EMA in 2020.^10^

The cephalosporin cefiderocol, while labile to hydrolysis from the common variants of NDM (NDM-1 and NDM-5),^11^ displays variable activity against organisms with this resistance mechanism due to its unique mode of entry into the cell.^12^ This entry is mediated by active transport through TonB-dependent siderophore uptake receptors such as CirA and Fiu.^13^ PBP3 is the preferential target of cefiderocol, and previously described insertions at amino acid position 333 in the *Escherichia coli* PBP3 have been directly shown to increase the MIC of cefiderocol 4-fold in an isogenic mutant strain,^14^ although the mutant strain in question was still susceptible to cefiderocol.

Aztreonam/avibactam is another combination of the monobactam aztreonam with a diazabicyclooctane β-lactamase inhibitor. Aztreonam/avibactam was approved by the EMA in 2024^15^ and the US FDA in 2025.^16^ While aztreonam/avibactam has demonstrated potent *in vitro* activity against MBL-positive Enterobacterales, the above-mentioned mutations in PBP3 in concert with production of select variants of plasmid-encoded AmpC (e.g. CMY-42 or CMY-145) have been shown to be a means of resistance to this combination in *E. coli.*^17^

Here we show the *in vitro* activity of aztreonam/nacubactam, cefepime/nacubactam and comparator agents, including cefiderocol and aztreonam/avibactam, against 630 carbapenemase-positive Enterobacterales isolates collected as part of a surveillance program from 2021 to 2023.

## Methods

Organisms were selected from among 12 613 clinical Enterobacterales isolates from a global surveillance study that included Asia and the South Pacific, Europe and North America in 2021–2023.^18^ Isolates that were non-susceptible to selected β-lactam agents, primarily meropenem (see Supplementary data available at *JAC-AMR* Online), were selected for molecular characterization. Of these, 630 carried one or more carbapenemase and are included in the current study.

Antimicrobial susceptibility testing was done by broth microdilution according to CLSI guidelines.^19^ Antimicrobial susceptibility was assessed using 2026 CLSI breakpoints^20^ or EUCAST breakpoints.^21^ Combinations were tested using a 1:1 ratio of nacubactam and β-lactam. Cefiderocol was tested using iron-depleted CAMHB. Quality control testing for each agent was performed according to CLSI guidelines^20^ on each day of testing; MIC values are reported only for results validated by this testing.

Molecular characterization was carried out using WGS by Promethion P2 Integrated (Oxford Nanopore Technologies, Oxford, UK). DNA isolation was carried out using a magnetic bead kit (Norgen, Toronto, Canada), library preparation was by rapid barcoding kit (SQK-RBK114.96) and libraries were sequenced using a P2 flow cell (R10.4.1). Base-calling was performed using dorado 7.6.7. Assembly was conducted using the EPI2ME isolates workflow version 1.4.1. Antimicrobial resistance markers were identified using AbritAMR.^22^ The deduced amino acid sequences of identified β-lactamase genes were extracted so that variants could be determined using the NCBI Bacterial Antimicrobial Resistance Reference Gene Database (Bioproject 313047). Mutations in the genes encoding PBP3, porins and TonB-dependent siderophore uptake receptors were assessed by pairwise alignment against the reference sequences using BLAST.^23^ MLSTs are reported for organisms for which schema are published.

## Results

### Resistance mechanisms

Of the 630 characterized isolates with carbapenemase genes, the majority (74%) were *Klebsiella pneumoniae* and a total of 307 isolates (49%) carried a variant of NDM (Table 1). Of these NDM-producing isolates, 87 also co-carried a gene encoding an OXA-48-like carbapenemase and 3 co-carried a different carbapenemase. Among the characterized isolates, 300 (48%) carried a serine-carbapenemase and no MBL. Approximately half of these carried a variant of KPC and the other half carried an OXA-48-like carbapenemase.

**Table 1.** Molecular mechanisms of resistance that impact β-lactam antibiotics in the studied isolates

Organism	MBL ± other β-lactamases					Serine-carbapenemase ± AmpC/ESBL				Porin mutations			Siderophore uptake mutations		PBP3 insertion	Grand total
	NDM	NDM + OXA-48-like	VIM	IMP	Mult.^a^	KPC	OXA-48-like	IMI	Mult.^b^	OmpC+F	OmpC	OmpF	CirA	Fiu		
*Klebsiella pneumoniae*	122	70	4	2	2	141	123		1	254	40	92				465
*Escherichia coli*	36	10		1	1	5	1			5	28	3	12	1	40	54
*Enterobacter* spp.	31	1	7	2		1	3	2			1	14	7	3		47
Morganellaceae	13	1	2		1	2	7				4		8	1		26
*Citrobacter* spp.	6		1		1	2						1				10
*Klebsiella* spp.	6	5		1		2	3		1	2	3	1	1	3		18
*Serratia* spp.	3		1			3	3					1				10
Grand total	217	87	15	6	5	156	140	2	2	261	76	112	28	8		630

The MBL and serine-carbapenemase categories are exclusive, the porin mutation, siderophore uptake mutations, and PBP3 insertion categories are non-exclusive with each other and the MBL and serine-carbapenemase categories.

^a^Mult.: multiple carbapenemases including an MBL: NDM-1+KPC-2 (*n* = 2); NDM-5+KPC-3 (*n* = 1); VIM-1+GES-6 (*n* = 1); VIM-1+OXA-48 (*n* = 1).

^b^Mult.: multiple serine-carbapenemases: KPC-2+OXA-181 (*n* = 1); KPC-3+OXA-48 (*n* = 1).

Of 54 *E. coli* that carried any carbapenemase, 40 (74%) also had a four amino acid insertion in PBP3 previously associated with β-lactam-resistance.^17^ Of the 47 isolates of *E. coli* producing NDM, 39 (83%) also had an insertion in PBP3. A complete list of the observed resistance mechanisms is in Table S1.

Of carbapenemase-producing isolates, 99.2% tested with MIC values ≤4/4 mg/L of aztreonam/nacubactam (Table 2). Cefepime/nacubactam was active against 96.7% of isolates at ≤8/8 mg/L. Other active agents were aztreonam/avibactam [97.1% susceptible (S) (CLSI and EUCAST)] and cefiderocol [91.9% S (CLSI), 72.4% S (EUCAST)] (Table 3).

**Table 2.** The percentage of Enterobacterales isolates inhibited by ≤4/4 mg/L aztreonam/nacubactam and ≤8/8 mg/L cefepime/nacubactam, by carbapenemase genotype

Carbapenemase genotype^a^	*n*	%ATM/NAC MIC ≤4/4 mg/L	%FEP/NAC MIC ≤8/8 mg/L
Any carbapenemase	630	99.2	96.7
NDM	307	98.7	94.1
NDM-1	174	98.9	92.5
NDM-4	12	100	100
NDM-5	115	98.3	95.7
VIM	17	94.1	82.4
VIM-1	14	100	85.7
KPC	158	100	100
KPC-2	89	100	100
KPC-3	61	100	100
OXA-48-like	142	100	100
OXA-48	73	100	100
OXA-232	44	100	100
OXA-181	14	100	100

^a^Isolates in the NDM category may carry additional serine-carbapenemases. Only genotypic subsets that contain 10 or more isolates are shown.

**Table 3.** The *in vitro* activity of aztreonam/nacubactam, cefepime/nacubactam and comparator agents against carbapenemase-producing Enterobacterales, by carbapenemase family

Agent	Genotype^a^ (number of isolates), MIC_50/90_ (mg/L), percent susceptible																			
	Any carbapenemase (630)				NDM (307)				VIM (17)				KPC (158)				OXA-48-like (142)			
	MIC_50_	MIC_90_	%Sus.		MIC_50_	MIC_90_	%Sus.		MIC_50_	MIC_90_	%Sus.		MIC_50_	MIC_90_	%Sus.		MIC_50_	MIC_90_	%Sus.	
			EUCAST	CLSI			EUCAST	CLSI			EUCAST	CLSI			EUCAST	CLSI			EUCAST	CLSI
Aztreonam/nacubactam	1	2	na	na	1	2	na	na	0.5	2	na	na	2	2	na	na	1	2	na	na
Aztreonam	>64	>64	5.4	8.1	>64	>64	6.5	8.8	32	>64	17.6	35.3	>64	>64	0.6	2.5	>64	>64	6.3	9.2
Cefepime/nacubactam	2	4	na	na	2	8	na	na	2	16	na	na	1	2	na	na	1	2	na	na
Cefepime	>64	>64	1.4	2.1	>64	>64	0.0	0.0	>64	>64	0.0	0.0	>64	>64	0.6	1.3	>64	>64	4.2	6.3
Aztreonam/avibactam	0.25	1	97.1	97.1	0.25	2	95.1	95.1	0.12	0.5	94.1	94.1	0.25	0.5	100	100	0.25	0.5	98.6	98.6
Ceftazidime/avibactam	>64	>64	46.7	46.7	>64	>64	2.0	2.0	>64	>64	0.0	0.0	1	4	96.2	96.2	1	2	95.8	95.8
Ceftazidime	>32	>32	1.4	2.9	>32	>32	0.3	0.3	>32	>32	0.0	0.0	>32	>32	0.0	2.5	>32	>32	5.6	9.2
Cefiderocol	2	4	72.4	91.9	2	8	55.0	85.7	1	8	82.4	88.2	1	2	94.9	98.7	1	4	83.1	97.9
Imipenem/relebactam	8	>32	34.9	26.8	32	>32	3.9	1.0	8	32	0.0	0.0	0.25	1	98.1	93.0	4	32	33.8	11.3
Imipenem	16	>32	5.6	1.9	32	>32	2.6	0.7	8	16	0.0	0.0	>32	>32	2.5	1.3	8	32	12.7	3.5
Meropenem/vaborbactam	16	>16	38.7	32.4	>16	>16	14.0	7.8	4	>16	76.5	58.8	0.5	4	97.5	91.1	>16	>16	19.0	12.7
Meropenem	>16	>16	6.0	2.5	>16	>16	2.6	0.7	4	>16	35.3	29.4	>16	>16	5.7	0.6	>16	>16	6.3	2.1
Piperacillin/tazobactam	>32	>32	0.5	0.5	>32	>32	0.0	0.0	>32	>32	0.0	0.0	>32	>32	0.6	0.6	>32	>32	0.7	0.7
Colistin	0.25	16	79.2	na	0.25	16	83.4	na	0.25	>16	76.5	na	0.25	16	82.9	na	0.25	>16	65.5	na

na, not available.

^a^Isolates in the MBL category may carry additional serine-carbapenemases. Only genotypic subsets that contain 10 or more isolates are shown.

### MBLs

NDM-1 and NDM-5 were the dominant variants of NDM in the organisms from this study. Against NDM-1-producing organisms, active agents were aztreonam/nacubactam (98.9% inhibited by ≤4/4 mg/L); cefepime/nacubactam (92.5% inhibited by ≤8/8 mg/L), aztreonam/avibactam [98.9% S (CLSI and EUCAST)] and cefiderocol [89.7% S (CLSI), 55.7% S (EUCAST)] (Table 4 and Figure S1). Isolates carrying NDM-5 were less frequently susceptible to aztreonam/avibactam [88.7% S (CLSI and EUCAST)] and cefiderocol [78.3% S (CLSI), 54.8% S (EUCAST)]; however, aztreonam/nacubactam and cefepime/nacubactam retained potency *in vitro*, with 98.3% and 95.7% testing with MIC values ≤4/4 mg/L and ≤8/8 mg/L, respectively.

**Table 4.** *In vitro* activity of aztreonam/nacubactam, cefepime/nacubactam and comparator agents against MBL-producing Enterobacterales, by variant

Agent	Genotype^a^ (number of isolates), MIC_50/90_ (mg/L), percent susceptible															
	NDM-1 (174)				NDM-4 (12)				NDM-5 (115)				VIM-1 (14)			
	MIC_50_	MIC_90_	%Sus.		MIC_50_	MIC_90_	%Sus.		MIC_50_	MIC_90_	%Sus.		MIC_50_	MIC_90_	%Sus.	
			EUCAST	CLSI			EUCAST	CLSI			EUCAST	CLSI			EUCAST	CLSI
Aztreonam/nacubactam	1	2	na	na	1	2	na	na	1	2	na	na	0.5	2	na	na
Aztreonam	>64	>64	6.3	8.6	>64	>64	0.0	8.3	>64	>64	7.8	9.6	16	>64	21.4	35.7
Cefepime/nacubactam	2	8	na	na	2	2	na	na	2	8	na	na	2	16	na	na
Cefepime	>64	>64	0.0	0.0	>64	>64	0.0	0.0	>64	>64	0.0	0.0	>64	>64	0.0	0.0
Aztreonam/avibactam	0.12	0.25	98.9	98.9	0.06	1	100	100	0.25	8	88.7	88.7	0.12	0.5	100	100
Ceftazidime/avibactam	>64	>64	1.1	1.1	>64	>64	16.7	16.7	>64	>64	1.7	1.7	>64	>64	0.0	0.0
Ceftazidime	>32	>32	0.0	0.0	>32	>32	0.0	0.0	>32	>32	0.9	0.9	>32	>32	0.0	0.0
Cefiderocol	2	8	55.7	89.7	2	4	50.0	91.7	2	32	54.8	78.3	1	4	85.7	92.9
Imipenem/relebactam	32	>32	4.0	0.6	32	>32	8.3	0.0	32	>32	3.5	1.7	8	32	0.0	0.0
Imipenem	32	>32	2.9	0.6	16	>32	0.0	0.0	32	>32	2.6	0.9	8	16	0.0	0.0
Meropenem/vaborbactam	>16	>16	18.4	9.8	>16	>16	0.0	0.0	>16	>16	9.6	6.1	4	>16	71.4	50.0
Meropenem	>16	>16	3.4	0.6	>16	>16	0.0	0.0	>16	>16	1.7	0.9	8	>16	35.7	28.6
Piperacillin/tazobactam	>32	>32	0.0	0.0	>32	>32	0.0	0.0	>32	>32	0.0	0.0	>32	>32	0.0	0.0
Colistin	0.25	>16	80.5	na	0.25	8	83.3	Na	0.25	16	87.0	na	0.25	>16	85.7	na

na, not available.

^a^Isolates in the MBL category may carry additional serine-carbapenemases. Only genotypic subsets that contain 10 or more isolates are shown.

**Table 5.** The *in vitro* activity of aztreonam/nacubactam, cefepime/nacubactam and comparator agents against serine-carbapenemase-producing Enterobacterales, by variant

Agent	Genotype^a^ (number of isolates), MIC_50/90_ (mg/L), percent susceptible																			
	KPC-2 (89)				KPC-3 (61)				OXA-48 (73)				OXA-232 (44)				OXA-181 (14)			
	MIC_50_	MIC_90_	%Sus.		MIC_50_	MIC_90_	%Sus.		MIC_50_	MIC_90_	%Sus.		MIC_50_	MIC_90_	%Sus.		MIC_50_	MIC_90_	%Sus.	
			EUCAST	CLSI			EUCAST	CLSI			EUCAST	CLSI			EUCAST	CLSI			EUCAST	CLSI
Aztreonam/nacubactam	2	2	na	na	2	2	na	na	2	2	na	na	1	2	na	na	1	2	na	na
Aztreonam	>64	>64	1.1	3.4	>64	>64	0.0	1.6	>64	>64	5.5	6.8	>64	>64	4.5	9.1	>64	>64	14.3	21.4
Cefepime/nacubactam	1	2	na	na	1	2	na	na	1	2	na	na	1	2	na	na	1	4	na	na
Cefepime	>64	>64	1.1	1.1	>64	>64	0.0	1.6	>64	>64	5.5	8.2	>64	>64	0.0	0.0	>64	>64	7.1	14.3
Aztreonam/avibactam	0.25	1	100	100	0.25	0.5	100	100	0.25	0.5	98.6	98.6	0.25	0.5	100	100	0.25	0.5	92.9	92.9
Ceftazidime/avibactam	1	2	97.8	97.8	2	4	100	100	1	2	98.6	98.6	1	2	97.7	97.7	1	>64	71.4	71.4
Ceftazidime	>32	>32	0.0	3.4	>32	>32	0.0	1.6	>32	>32	5.5	8.2	>32	>32	2.3	9.1	>32	>32	14.3	14.3
Cefiderocol	1	2	94.4	98.9	1	2	98.4	100	1	4	78.1	95.9	1	2	90.9	100	1	4	78.6	100
Imipenem/relebactam	0.25	1	98.9	94.4	0.25	0.5	100	95.1	4	>32	24.7	9.6	4	8	45.5	15.9	4	>32	28.6	0.0
Imipenem	32	>32	2.2	1.1	>32	>32	0.0	0.0	8	>32	9.6	4.1	8	16	15.9	2.3	8	>32	0.0	0.0
Meropenem/vaborbactam	0.25	8	97.8	88.8	0.5	1	100	100	16	>16	21.9	16.4	>16	>16	11.4	9.1	16	>16	14.3	7.1
Meropenem	>16	>16	3.4	1.1	>16	>16	6.6	0.0	>16	>16	9.6	4.1	>16	>16	2.3	0.0	>16	>16	7.1	0.0
Piperacillin/tazobactam	>32	>32	1.1	1.1	>32	>32	0.0	0.0	>32	>32	1.4	1.4	>32	>32	0.0	0.0	>32	>32	0.0	0.0
Colistin	0.25	>16	80.9	na	0.25	8	86.9	na	0.5	>16	58.9	na	0.25	16	75.0	na	0.25	>16	64.3	na

na, not available.

^a^Isolates that carry MBLs are not included in any of these genotypic subgroups. Two isolates co-carry KPC and OXA-48-like enzymes. They are present in the KPC and the OXA-48-like categories.

While few isolates carried an MBL other than NDM, 14/17 VIM-positive isolates carried VIM-1. Of these, 100% were inhibited by ≤4/4 mg/L aztreonam/nacubactam and were susceptible to aztreonam/avibactam (CLSI and EUCAST) and 85.7% were inhibited by ≤8/8 mg/L cefepime/nacubactam. Cefiderocol also retained activity against VIM-1-positive isolates [92.9% S (CLSI), 85.7% S (EUCAST)].

### Serine-carbapenemases

Of 158 KPC-positive isolates, 89 carried KPC-2 and 61 carried KPC-3. Both aztreonam/nacubactam and cefepime/nacubactam were active against all isolates that carried KPC-2 or KPC-3, as was aztreonam/avibactam (Table 3 and Figure S2). Other agents that demonstrated potent *in vitro* activity against KPC-producing isolates were: ceftazidime/avibactam [96.2% S (CLSI and EUCAST)], cefiderocol [98.7% S (CLSI), 94.9% S (EUCAST)], imipenem/relebactam [93.0% S (CLSI), 98.1% S (EUCAST)] and meropenem/vaborbactam [91.1% S (CLSI), 97.5% S (EUCAST)]. Regarding meropenem/vaborbactam, fewer KPC-2-positive isolates were susceptible than KPC-3-positive isolates, 88.8% S (CLSI) versus 100% S (CLSI), respectively.

Six isolates were characterized that produced variants of KPC, no MBL and were resistant to ceftazidime/avibactam (Table S5). The MIC values of aztreonam/nacubactam against these isolates ranged from 0.12/0.12 to 2/2 mg/L (mode: 1/1 mg/L) and the MIC values of cefepime/nacubactam ranged from 1/1 to 8/8 mg/L (mode: 1/1 mg/L).

Of 142 isolates that carried a gene encoding an OXA-48-like carbapenemase, 73 carried OXA-48, 44 carried OXA-232, 14 carried OXA-181 and the remaining 11 isolates carried other variants. Aztreonam/nacubactam and cefepime/nacubactam were the only agents tested that were active against 100% of the isolates that carried OXA-48-like carbapenemases (using the surrogate breakpoints of ≤4/4 mg/L and ≤8/8 mg/L, respectively). Other agents that were active against organisms positive for an OXA-48-like carbapenemase were: aztreonam/avibactam [98.6% S (CLSI and EUCAST)], ceftazidime/avibactam [95.8% S (CLSI and EUCAST)] and cefiderocol [97.9% S (CLSI), 83.1% S (EUCAST)].

### NDM and OXA-48-like

**Figure 1.**
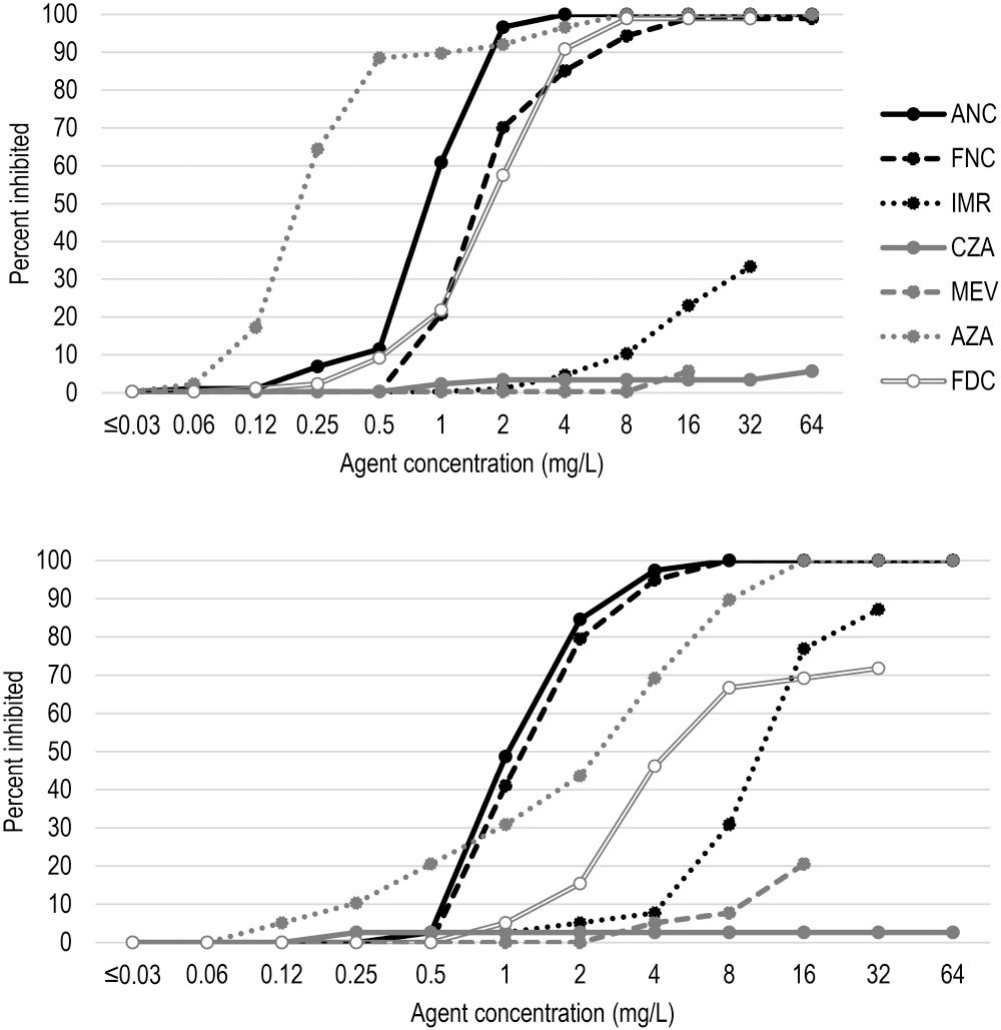
Cumulative percentage of isolates inhibited by increasing concentrations of agents that (top) co-produce NDM and OXA-48-like carbapenemases (*n* = 87) or (bottom) produce NDM and carry an insertion in PBP3 (*n* = 39). ANC, aztreonam/nacubactam; AZA, aztreonam/avibactam; CZA, ceftazidime/avibactam; FDC, cefiderocol; FNC, cefepime/nacubactam; IMR, imipenem/relebactam; MEV, meropenem/vaborbactam.

Among the 307 NDM-positive isolates in this study, 87 (28%) co-carried a variant of OXA of the OXA-48-like subfamily. All of these were inhibited by aztreonam/nacubactam at ≤4/4 mg/L, and 94.3% were inhibited by cefepime/nacubactam at ≤8/8 mg/L (Figure 1). The only other agents that demonstrated *in vitro* activity were aztreonam/avibactam [96.6% S (CLSI and EUCAST)] and cefiderocol [90.8% S (CLSI), 57.5% S (EUCAST)].

### Insertions in PBP3 among carbapenemase-positive E. coli

Of the 39 isolates with a PBP3 insertion that carried a variant of NDM, 97.4% were inhibited by aztreonam/nacubactam at ≤4/4 mg/L and 100% were inhibited by cefepime/nacubactam at ≤8/8 mg/L (Figure 1). Susceptibility to other agents that are often active against MBL-producing Enterobacterales was less frequent against this population, with 69.2% S (CLSI and EUCAST) to aztreonam/avibactam, and 46.2% S (CLSI) and 15.4% S (EUCAST) to cefiderocol (Figure 1). The isolates of *E. coli* in this study were epidemiologically diverse, belonging to >25 different STs (Table S1).

**Figure 2.**
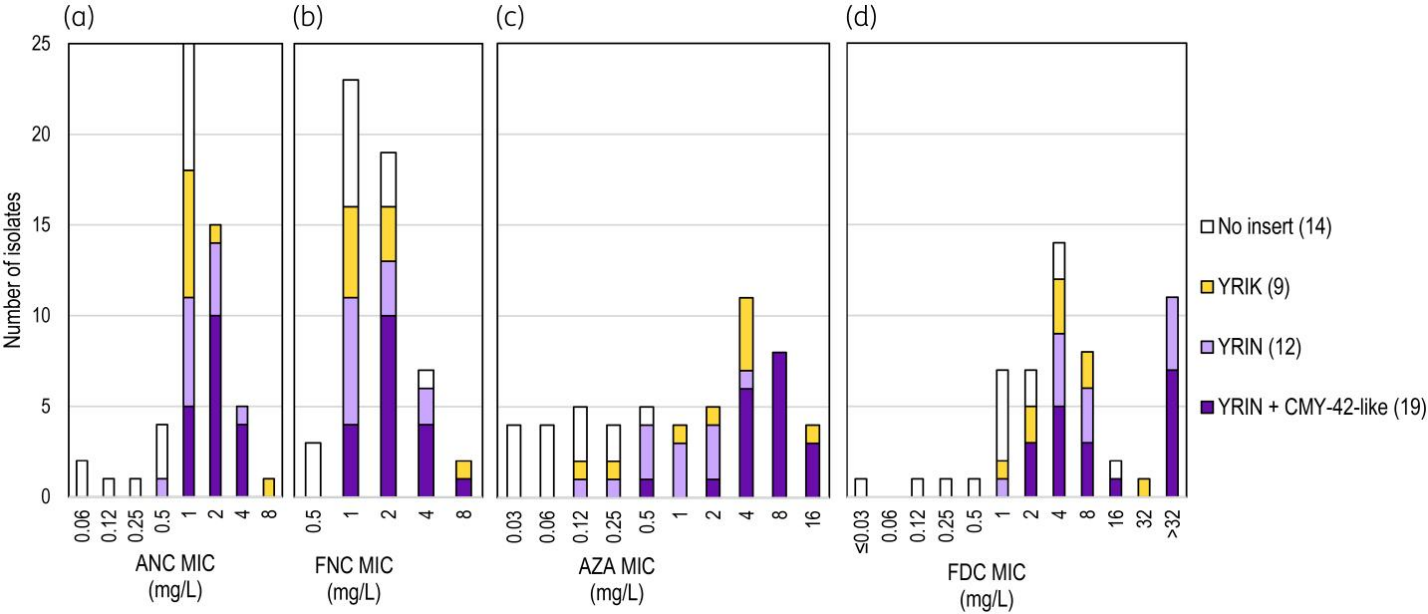
MIC distribution of (a) aztreonam/nacubactam, (b) cefepime/nacubactam, (c) aztreonam/avibactam and (d) cefiderocol against carbapenemase-positive *E. coli* isolates, by PBP3 insert and presence of CMY-42-like AmpC. ANC, aztreonam/nacubactam; AZA, aztreonam/avibactam; FDC, cefiderocol; FNC, cefepime/nacubactam.

The presence of an insertion in PBP3 with or without a CMY-42-like enzyme among carbapenemase-positive *E. coli* isolates was not associated with a change in the modal MIC of aztreonam/nacubactam or cefepime/nacubactam (modal MICs of 1–2 mg/L regardless of genotype) (Figure 2). This was not true for aztreonam/avibactam with isolates that carried Tyr-Arg-Ile-(Lys/Asn) insertions at position 333, demonstrating higher MIC values for the combination than those without, and isolates that carried Tyr-Arg-Ile-Asn insertions + CMY-42-like enzymes demonstrating modal aztreonam/avibactam MIC values of 8 mg/L (resistant using EUCAST breakpoints). Insertions were present in >90% of cefiderocol-non-susceptible, carbapenemase-positive *E. coli* isolates (21/22, CLSI breakpoints; 33/36, EUCAST breakpoints). The carriage of CMY-42-like β-lactamases in isolates with Tyr-Arg-Ile-Asn inserts in PBP3 was not associated with a difference in MIC distribution for cefiderocol when compared with isolates that did not carry CMY-42-like enzymes.

### Siderophore uptake mutants and porin deficiencies

The *in vitro* activity of aztreonam/nacubactam, cefepime/nacubactam and comparator agents against cefiderocol-resistant isolates that have mutations in genes encoding TonB-dependent siderophore uptake receptors (*cirA* and/or *fiu*) is shown in Table S3. Both aztreonam/nacubactam and cefepime/nacubactam were active against all 27 cefiderocol-resistant isolates that encoded a premature stop codon in either *cirA* or *fiu* (MIC_90_ values of 2/2 mg/L for aztreonam/nacubactam and 4/4 mg/L for cefepime/nacubactam). The MIC_90_ value for aztreonam/avibactam was 8 mg/L and 85.2% were susceptible.

The *in vitro* activity of aztreonam/nacubactam, cefepime/nacubactam and comparator agents against isolates stratified by deficiencies in porins OmpC and OmpF and their homologues is shown in Table S4. The MIC_50_ and MIC_90_ values of aztreonam/nacubactam and cefepime/nacubactam against isolates with any combination of deleterious mutations in OmpC and OmpF were within one dilution of the values against isolates with no mutation in the genes encoding these porins (aztreonam/nacubactam MIC_50_: 1/1–2/2 mg/L, MIC_90_: 2/2 mg/L; cefepime/nacubactam MIC_50_: 2/2 mg/L, MIC_90_: 4/4–8/8 mg/L).

## Discussion

This study shows the *in vitro* activity of two novel β-lactam/β-lactamase inhibitor combinations: aztreonam and cefepime with nacubactam, against 630 carbapenemase-positive Enterobacterales isolates collected globally from 2021 to 2023.

Both aztreonam and cefepime when combined with nacubactam inhibited every isolate that carried either a KPC or OXA-48-like carbapenemase at concentrations below the CLSI breakpoints for the β-lactam agents on their own.

Aztreonam/nacubactam demonstrated potent *in vitro* activity against isolates that carry MBLs while cefepime/nacubactam demonstrated potency similar to aztreonam/avibactam.

Isolates that carry NDM-5 comprised over one-third of the NDM-producing organisms in this study. Aztreonam/avibactam has been previously reported to be active less frequently against NDM-5-producing Enterobacterales isolates than NDM-1-producing Enterobacterales through mechanisms independent of the variant of NDM.^17^ This phenomenon was supported in this study as well. This could be due to co-carriage with other β-lactamases such as CMY-42 and CMY-145, which are AmpC with mutations that enhance catalysis of cephalosporins and aztreonam and have previously been implicated in aztreonam/avibactam resistance.^17^ In the present study, CMY-145 (*n* = 9) and CMY-42 (*n* = 9) were both observed only in isolates that produced NDM-5, while variants of CMY co-produced in NDM-1-positive isolates were not among those previously associated with aztreonam/avibactam resistance (CMY-4, *n* = 7; CMY-16, *n* = 5; CMY-6, *n* = 4; and CMY-2, *n* = 1). Notably, the percentages of NDM-5-producing isolates that were inhibited by aztreonam/nacubactam at ≤4/4 mg/L or by cefepime/nacubactam at ≤8/8 mg/L were very similar to those that produce NDM-1.

Another threat on the rise is the co-production of NDM and OXA-48-like carbapenemases. Aztreonam/nacubactam showed the greatest potency against the 87 isolates that carried these carbapenemases, with 100% inhibited by ≤4/4 mg/L.

One other resistance mechanism that threatens the activity of relatively new therapeutics such as cefiderocol and aztreonam/avibactam is a four amino acid insertion in PBP3 at or adjacent to position 333.^14,17,24^ Multiple *in vitro* studies have shown that this mechanism must work in concert with specific β-lactamases to increase aztreonam/avibactam or cefiderocol MIC values above resistance breakpoints. In the present study, 81% of NDM-producing *E. coli* isolates carried such an insertion in PBP3. Long *et al.*^25^ studied available genome sequences of *E. coli* isolates and found that 83% of isolates with PBP3 insertions also carry a variant of NDM. Taken together, our data indicate that there is a strong bi-directional linkage between NDM carriage and insertions in PBP3, with one mechanism frequently seen alongside the other in clinically isolated *E. coli*. Of the agents tested, aztreonam/nacubactam and cefepime/nacubactam both retained potent *in vitro* activity, while both cefiderocol and aztreonam/avibactam activity was far lower than it was against NDM-positive Enterobacterales with no PBP3 insertion, which were primarily *Klebsiella pneumoniae*. This study shows an alarming incidence of chromosomal resistance mechanisms that impact aztreonam/avibactam and cefiderocol, particularly among recently collected clinical isolates of *E. coli*. This is concerning given that cefiderocol has only been available for clinical use since 2019, two years before the start of collection for this study, and aztreonam/avibactam has only been available for clinical use since 2024, after the collection window for this study. It is notable that ceftazidime/avibactam has been clinically available since 2015, and co-administration of ceftazidime/avibactam and aztreonam well pre-dates the approval of aztreonam/avibactam.^26^ Future work to enhance our understanding of the clinical prevalence of PBP3 mutations in *E. coli* and the therapeutic regimens that select for these mutations may be critical to understanding and controlling the spread of this mechanism of resistance, which is of renewed importance with the recent and likely upcoming approvals of novel β-lactam/β-lactamase inhibitor combinations that rely primarily on the inhibition of PBP3.

One limitation of *in vitro* studies comparing the activity of β-lactamase inhibitors that have antimicrobial activity, such as the diazabicyclooctanes nacubactam and avibactam, resides in the way they are tested. CLSI guidelines for avibactam are to test at a fixed 4 mg/L with the β-lactam antibiotic and testing with nacubactam is done at a fixed 1:1 ratio with the β-lactam antibiotic. This can cause discrepancies in the comparison of their *in vitro* activities, demonstrating high potency for aztreonam/avibactam at low concentrations of aztreonam, where synergy with 4 mg/L of avibactam may play a role in potency. A relatively greater activity for the nacubactam combinations may be observed when testing concentrations ≥8 mg/L as the antibacterial activity of nacubactam may start to exceed that of the β-lactam companion in isolates with poorly inhibited β-lactamases.

Aztreonam/nacubactam and cefepime/nacubactam show potent *in vitro* activity against MBL-positive *E. coli* isolates with PBP3 mutations, likely owing to the PBP2-inhibitory activity of nacubactam. These combinations, which are not reliant on inhibition of PBP3 or entry through siderophore uptake mechanisms, present promising combinations for continued development as therapeutic options against organisms that are rapidly becoming resistant to agents that have only briefly been utilized clinically.

## Supplementary Material

dlag166_Supplementary_Data

## Supplementary data

Figures S1 and S2 and Tables S1–S5 are available as Supplementary data at *JAC-AMR* Online.
