# Supplementary material for: In vitro activities of aztreonam/nacubactam and cefepime/nacubactam against a global collection of molecularly characterized Enterobacterales isolates carrying carbapenemases: a global antimicrobial resistance surveillance study from 2021 to 2023
Source: JAC Antimicrob Resist. 2026 Aug 12;8(4):dlag166. doi: 10.1093/jacamr/dlag166 (PMC13463764; doi:10.1093/jacamr/dlag166)
Supplement: Supplementary file 1 [file dlag166_supplementary_data.docx]

Supplementary methods:

Isolates were those from Karlowsky *et al.* *JAC Antimicrob Resist.* 2026;**8** that met one of the following criteria using CLSI breakpoints: ATM/NAC MIC ≥4 µg/mL, FEP/NAC MIC ≥4 µg/mL, AZA-NS, CZA-NS, FDC-NS, MEM-NS, MEM-VAB-NS. This led to characterization of 785 isolates. 630 isolates in this population carried genes encoding carbapenemases and are shown here.

Supplementary Table 1. β-lactam-resistance mechanisms in the studied isolates

| Organism, carbapenemase (hierarchy) | Carbapenemases (family) | Carbapenemases (variants) | Porin mutations | PBP3 insertion | Siderophore uptake | n |
| --- | --- | --- | --- | --- | --- | --- |
| ***Citrobacter* spp.** |  |  |  |  |  | **10** |
| MBL ± others | NDM + KPC | NDM-1 + KPC-2 | N | N | N | 1 |
|  | NDM | NDM-1 | N | N | N | 4 |
|  |  | NDM-5 | N | N | N | 1 |
|  |  | NDM-7 | N | N | N | 1 |
|  | VIM | VIM-1 | OmpF-mut | N | N | 1 |
| Serine-carbapenemase ± aAmpC ± ESBL | KPC | KPC-2 | N | N | N | 2 |
| ***Enterobacter* spp.** |  |  |  |  |  | **47** |
| MBL ± others | IMP | IMP-8 | N | N | N | 2 |
|  | NDM | NDM-1 | N | N | N | 15 |
|  |  |  | N | N | CirA-LoF | 5 |
|  |  |  | OmpF-mut | N | CirA-LoF | 1 |
|  |  |  | N | N | Fiu-LoF | 1 |
|  |  | NDM-4 | OmpF-mut | N | N | 2 |
|  |  |  | N | N | N | 1 |
|  |  | NDM-5 | N | N | N | 5 |
|  |  |  | OmpF-mut | N | N | 1 |
|  | NDM + OXA-48-like | NDM-1 + OXA-181 | OmpF-mut | N | CirA-LoF | 1 |
|  | VIM | VIM-1 | N | N | N | 3 |
|  |  |  | OmpC-mut | N | N | 1 |
|  |  |  | OmpF-mut | N | N | 1 |
|  |  |  | N | N | Fiu-LoF | 1 |
|  |  | VIM-4 | N | N | Fiu-LoF | 1 |
| Serine-carbapenemase ± aAmpC ± ESBL | IMI | IMI-1 | N | N | N | 1 |
|  |  | IMI-4 | OmpF-mut | N | N | 1 |
|  | KPC | KPC-3 | N | N | N | 1 |
|  | OXA-48-like | OXA-48 | OmpF-mut | N | N | 3 |
| ***Escherichia coli*** |  |  |  |  |  | **54** |
| MBL ± others | IMP | IMP-8 | OmpC-mut | N | N | 1 |
|  | NDM | NDM-1 | OmpF-mut | N | N | 1 |
|  |  |  | N | N | N | 1 |
|  |  | NDM-5 | N | 333INS "YRIN" | CirA-LoF | 9 |
|  |  |  | N | N | N | 5 |
|  |  |  | N | 333INS "YRIN" | N | 5 |
|  |  |  | N | N | N | 5 |
|  |  |  | OmpC/F-mut | 333INS "YRIK" | N | 3 |
|  |  |  | OmpC-mut | 333INS "YRIK" | N | 3 |
|  |  |  | N | 333INS "YRIN" | N | 1 |
|  |  |  | OmpF-mut | 333INS "YRIN" | N | 1 |
|  |  |  | N | 333INS "YRIK" | Fiu-LoF | 1 |
|  |  | NDM-74 | OmpC-mut | N | N | 1 |
|  | NDM + KPC | NDM-5 + KPC-3 | OmpC-mut | 333INS "YRIN" | N | 1 |
|  | NDM + OXA-48-like | NDM-1 + OXA-244 | OmpC-mut | 333INS "YRIN" | N | 1 |
|  |  | NDM-5 + OXA-181 | OmpC/F-mut | 333INS "YRIN" | N | 1 |
|  |  | NDM-5 + OXA-181 | OmpC-mut | 333INS "YRIN" | CirA-LoF | 1 |
|  |  | NDM-5 + OXA-181 | N | N | N | 7 |
| Serine-carbapenemase ± aAmpC ± ESBL | KPC | KPC-2 | OmpF-mut | N | N | 1 |
|  |  |  | N | N | N | 3 |
|  |  | KPC-3 | N | N | CirA-LoF | 1 |
|  | OXA-48-like | OXA-244 | OmpC-mut | 333INS "YRIN" | CirA-LoF | 1 |
| ***Klebsiella pneumoniae*** |  |  |  |  |  | 465 |
| MBL ± others | IMP | IMP-8 | N | N | N | 2 |
|  | NDM | NDM-1 | OmpC/F-mut | N | N | 16 |
|  |  |  | OmpC-mut | N | N | 1 |
|  |  |  | OmpF-mut | N | N | 44 |
|  |  |  | N | N | N | 27 |
|  |  | NDM-1 + NDM-5 | OmpC-mut | N | N | 1 |
|  |  | NDM-4 | OmpF-mut | N | N | 2 |
|  |  |  | N | N | N | 3 |
|  |  | NDM-5 | OmpC/F-mut | N | N | 15 |
|  |  |  | OmpC-mut | N | N | 2 |
|  |  |  | OmpF-mut | N | N | 2 |
|  |  |  | N | N | N | 7 |
|  |  | NDM-7 | OmpF-mut | N | N | 2 |
|  | Multiple/other | NDM-1 + KPC-2 | OmpF-mut | N | N | 1 |
|  |  | VIM-1 + GES-6 | N | N | N | 1 |
|  | NDM + OXA-48-like | NDM-1 + NDM-5 + OXA-232 | OmpC/F-mut | N | N | 1 |
|  |  | NDM-1 + OXA-232 | OmpC/F-mut | N | N | 14 |
|  |  | NDM-1 + OXA-48 | OmpC/F-mut | N | N | 6 |
|  |  |  | OmpC-mut | N | N | 4 |
|  |  |  | OmpF-mut | N | N | 5 |
|  |  | NDM-4 + OXA-181 | OmpC/F-mut | N | N | 2 |
|  |  | NDM-4 + OXA-181 + OXA-48-NV | OmpC/F-mut | N | N | 1 |
|  |  | NDM-5 + OXA-181 | OmpC/F-mut | N | N | 16 |
|  |  | NDM-5 + OXA-232 | OmpC/F-mut | N | N | 14 |
|  |  |  | OmpC-mut | N | N | 3 |
|  |  |  | N | N | N | 1 |
|  |  | NDM-5 + OXA-48 | OmpC/F-mut | N | N | 3 |
|  | VIM | VIM-1 | N | N | N | 4 |
| Serine-carbapenemase ± aAmpC ± ESBL | KPC | KPC-17 | OmpC/F-mut | N | N | 1 |
|  |  | KPC-2 | OmpC/F-mut | N | N | 37 |
|  |  |  | OmpC-mut | N | N | 3 |
|  |  |  | OmpF-mut | N | N | 26 |
|  |  |  | N | N | N | 12 |
|  |  | KPC-2 + KPC-97 | OmpC/F-mut | N | N | 1 |
|  |  | KPC-3 | OmpC/F-mut | N | N | 39 |
|  |  |  | OmpC-mut | N | N | 2 |
|  |  |  | OmpF-mut | N | N | 3 |
|  |  |  | N | N | N | 14 |
|  |  | KPC-31 | OmpC/F-mut | N | N | 1 |
|  |  | KPC-49 | OmpC/F-mut | N | N | 2 |
|  | KPC+OXA-48-like | KPC-3 + OXA-48 | OmpC/F-mut | N | N | 1 |
|  | OXA-48-like | OXA-1307 | OmpC/F-mut | N | N | 1 |
|  |  | OXA-181 | OmpC/F-mut | N | N | 10 |
|  |  |  | OmpC-mut | N | N | 2 |
|  |  |  | N | N | N | 1 |
|  |  | OXA-232 | OmpC/F-mut | N | N | 33 |
|  |  |  | OmpC-mut | N | N | 7 |
|  |  |  | OmpF-mut | N | N | 1 |
|  |  |  | N | N | N | 2 |
|  |  | OXA-232 + OXA-48-New variant | OmpC/F-mut | N | N | 1 |
|  |  | OXA-244 | OmpC/F-mut | N | N | 3 |
|  |  | OXA-48 | OmpC/F-mut | N | N | 36 |
|  |  |  | OmpC-mut | N | N | 13 |
|  |  |  | OmpF-mut | N | N | 6 |
|  |  |  | N | N | N | 5 |
|  |  | OXA-48-Type | OmpC-mut | N | N | 2 |
| ***Klebsiella* spp.** |  |  |  |  |  | **18** |
| MBL ± others | IMP | IMP-8 | N | N | N | 1 |
|  | NDM | NDM-1 | OmpF-mut | N | N | 1 |
|  |  |  | N | N | N | 4 |
|  |  | NDM-5 | OmpC/F-mut | N | N | 1 |
|  | NDM + OXA-48-like | NDM-1 + OXA-48 | N | N | Fiu-LoF | 3 |
|  |  | NDM-4 + OXA-181 | OmpC-mut | N | N | 1 |
|  |  | NDM-5 + OXA-181 | OmpC/F-mut | N | N | 1 |
| Serine-carbapenemase ± aAmpC ± ESBL | KPC | KPC-2 | N | N | N | 1 |
|  |  | KPC-6 | N | N | N | 1 |
|  | KPC + OXA-48-like | KPC-2 + OXA-181 | OmpC-mut | N | N | 1 |
|  | OXA-48-like | OXA-232 | N | N | N | 1 |
|  |  | OXA-48 | OmpC-mut | N | N | 1 |
|  |  |  | N | N | CirA-LoF | 1 |
| ***Morganellaceae*** |  |  |  |  |  | **26** |
| MBL ± others | NDM | NDM-1 | OmpC-mut | N | N | 4 |
|  |  |  | N | N | Fiu-LoF | 1 |
|  |  |  | N | N | N | 8 |
|  | NDM + OXA-48-like | NDM-1 + OXA-181 | N | N | N | 1 |
|  | VIM | VIM-4 + VIM-75 | N | N | N | 1 |
|  |  | VIM-78 | N | N | N | 1 |
|  | VIM + OXA-48-like | VIM-1 + OXA-48 | N | N | N | 1 |
| Serine-carbapenemase ± aAmpC ± ESBL | KPC | KPC-2 | N | N | CirA-LoF | 2 |
|  | OXA-48-like | OXA-181 | N | N | N | 1 |
|  |  | OXA-48 | N | N | CirA-LoF | 6 |
| ***Serratia* spp.** |  |  |  |  |  | **10** |
| MBL ± others | NDM | NDM-1 | N | N | N | 3 |
|  | VIM | VIM-1 | N | N | N | 1 |
| Serine-carbapenemase ± aAmpC ± ESBL | KPC | KPC-2 | N | N | N | 2 |
|  |  | KPC-3 | OmpF-mut | N | N | 1 |
|  | OXA-48-like | OXA-1308 | N | N | N | 1 |
|  |  | OXA-48 | N | N | N | 2 |
| **Grand Total** |  |  |  |  |  | **630** |

Supplementary Table 2. Multilocus sequence types of studied isolates, by carbapenemase carriage

| **Organism, MLST** | **NDM** | **NDM; OXA-48-like** | **VIM** | **IMP** | **KPC** | **OXA-48-like** | **IMI** | **Multiple/other** | **Grand Total** |
| --- | --- | --- | --- | --- | --- | --- | --- | --- | --- |
| **Citrobacter spp.** | **6** |  | **1** |  | **2** |  |  | **1** | **10** |
| 18 | 1 |  |  |  |  |  |  |  | 1 |
| 154 |  |  | 1 |  |  |  |  |  | 1 |
| 169 | 1 |  |  |  |  |  |  | 1 | 2 |
| 335 | 1 |  |  |  |  |  |  |  | 1 |
| 511 | 1 |  |  |  |  |  |  |  | 1 |
| 862 |  |  |  |  | 1 |  |  |  | 1 |
| Novel | 2 |  |  |  | 1 |  |  |  | 3 |
| **Enterobacter spp.** | **31** | **1** | **7** | **2** | **1** | **3** | **2** |  | **47** |
| 45 | 1 |  |  |  |  |  |  |  | 1 |
| 78 | 1 |  |  | 1 |  | 2 |  |  | 4 |
| 90 |  |  |  | 1 |  |  |  |  | 1 |
| 91 | 2 |  |  |  |  |  |  |  | 2 |
| 102 |  |  | 1 |  |  |  |  |  | 1 |
| 114 | 4 |  |  |  |  |  |  |  | 4 |
| 133 |  |  | 1 |  |  |  |  |  | 1 |
| 134 | 5 |  |  |  |  |  |  |  | 5 |
| 151 |  |  |  |  |  | 1 |  |  | 1 |
| 171 | 5 |  |  |  |  |  |  |  | 5 |
| 177 | 2 |  |  |  |  |  |  |  | 2 |
| 200 | 1 |  |  |  |  |  |  |  | 1 |
| 265 |  |  | 1 |  |  |  |  |  | 1 |
| 269 |  |  |  |  | 1 |  |  |  | 1 |
| 418 | 1 |  |  |  |  |  |  |  | 1 |
| 462 |  |  | 1 |  |  |  |  |  | 1 |
| 544 | 3 |  |  |  |  |  |  |  | 3 |
| 662 |  |  | 1 |  |  |  |  |  | 1 |
| 937 | 1 |  |  |  |  |  |  |  | 1 |
| 1325 |  | 1 |  |  |  |  |  |  | 1 |
| 1540 | 2 |  |  |  |  |  |  |  | 2 |
| 1592 |  |  |  |  |  |  | 1 |  | 1 |
| 1718 | 2 |  |  |  |  |  |  |  | 2 |
| Novel | 1 |  | 2 |  |  |  | 1 |  | 4 |
| **Escherichia coli** | **36** | **10** |  | **1** | **5** | **1** |  | **1** | **54** |
| 46 | 3 |  |  |  |  |  |  |  | 3 |
| 101 |  | 1 |  |  |  |  |  |  | 1 |
| 107 | 1 |  |  |  |  |  |  |  | 1 |
| 131 | 2 |  |  |  | 3 |  |  |  | 5 |
| 141 |  |  |  |  | 1 |  |  |  | 1 |
| 155 | 1 |  |  |  |  |  |  |  | 1 |
| 156 | 1 |  |  |  |  |  |  |  | 1 |
| 167 | 7 | 2 |  |  |  | 1 |  |  | 10 |
| 224 | 1 |  |  |  |  |  |  |  | 1 |
| 361 | 2 | 2 |  |  |  |  |  | 1 | 5 |
| 393 | 1 |  |  |  |  |  |  |  | 1 |
| 405 | 4 |  |  |  |  |  |  |  | 4 |
| 410 | 2 |  |  |  |  |  |  |  | 2 |
| 448 |  | 1 |  |  |  |  |  |  | 1 |
| 457 |  |  |  | 1 |  |  |  |  | 1 |
| 617 | 2 |  |  |  |  |  |  |  | 2 |
| 648 | 2 |  |  |  |  |  |  |  | 2 |
| 1193 |  |  |  |  | 1 |  |  |  | 1 |
| 1284 | 1 |  |  |  |  |  |  |  | 1 |
| 1702 | 1 |  |  |  |  |  |  |  | 1 |
| 2083 | 1 |  |  |  |  |  |  |  | 1 |
| 2851 |  | 2 |  |  |  |  |  |  | 2 |
| 7036 | 1 |  |  |  |  |  |  |  | 1 |
| 11021 | 1 |  |  |  |  |  |  |  | 1 |
| 11681 | 1 |  |  |  |  |  |  |  | 1 |
| Novel | 1 | 2 |  |  |  |  |  |  | 3 |
| **Klebsiella pneumoniae** | **122** | **70** | **4** | **2** | **141** | **123** |  | **3** | **465** |
| 11 | 17 | 3 |  |  | 12 | 6 |  |  | 38 |
| 14 |  | 1 |  |  |  |  |  |  | 1 |
| 15 | 3 |  |  |  | 20 | 1 |  |  | 24 |
| 16 | 4 | 15 |  |  | 3 | 12 |  |  | 34 |
| 17 | 4 |  |  | 1 |  |  |  |  | 5 |
| 23 |  |  |  |  |  | 10 |  |  | 10 |
| 35 |  |  |  |  | 1 |  |  | 1 | 2 |
| 39 |  |  |  |  | 8 |  |  |  | 8 |
| 43 | 1 |  |  |  |  |  |  |  | 1 |
| 45 | 1 |  |  |  | 1 |  |  |  | 2 |
| 70 | 1 |  |  |  |  |  |  |  | 1 |
| 101 | 4 | 1 |  |  | 35 | 12 |  |  | 52 |
| 111 | 1 |  |  |  | 1 |  |  |  | 2 |
| 147 | 51 | 26 |  |  | 3 | 12 |  |  | 92 |
| 219 | 1 |  |  |  |  |  |  |  | 1 |
| 231 |  | 1 |  |  |  | 26 |  |  | 27 |
| 234 |  |  |  |  | 1 |  |  |  | 1 |
| 258 |  |  |  |  | 18 |  |  | 2 | 20 |
| 277 |  |  | 1 |  |  |  |  |  | 1 |
| 290 |  |  | 1 |  |  |  |  |  | 1 |
| 307 | 5 |  | 1 |  | 27 | 1 |  |  | 34 |
| 323 |  |  |  |  | 2 |  |  |  | 2 |
| 340 | 1 |  |  |  |  | 3 |  |  | 4 |
| 348 |  |  |  |  | 1 |  |  |  | 1 |
| 391 | 1 |  |  |  |  |  |  |  | 1 |
| 392 |  |  |  |  | 1 | 1 |  |  | 2 |
| 395 | 8 | 11 |  |  |  | 13 |  |  | 32 |
| 437 | 2 | 3 |  |  |  | 11 |  |  | 16 |
| 488 | 1 |  |  |  |  |  |  |  | 1 |
| 512 |  |  |  |  | 3 |  |  |  | 3 |
| 656 | 3 | 1 |  |  |  |  |  |  | 4 |
| 664 | 1 |  |  |  |  |  |  |  | 1 |
| 745 |  |  |  |  | 1 |  |  |  | 1 |
| 776 | 1 |  |  |  |  |  |  |  | 1 |
| 881 |  |  |  | 1 |  |  |  |  | 1 |
| 2096 |  | 1 |  |  |  | 9 |  |  | 10 |
| 2355 | 1 |  |  |  |  |  |  |  | 1 |
| 2451 |  |  | 1 |  |  |  |  |  | 1 |
| 2497 |  |  |  |  |  | 3 |  |  | 3 |
| 2657 | 1 |  |  |  |  |  |  |  | 1 |
| 3227 |  |  |  |  |  | 1 |  |  | 1 |
| 4843 |  |  |  |  |  | 1 |  |  | 1 |
| 5351 | 1 |  |  |  |  |  |  |  | 1 |
| 5859 | 1 | 4 |  |  |  |  |  |  | 5 |
| 5912 | 1 |  |  |  |  |  |  |  | 1 |
| 6260 | 6 | 2 |  |  |  |  |  |  | 8 |
| Novel |  | 1 |  |  | 3 | 1 |  |  | 5 |
| **Klebsiella spp.** | **6** | **5** |  | **1** | **2** | **3** |  | **1** | **18** |
| 4 |  | 1 |  |  | 1 | 1 |  | 1 | 4 |
| 9 |  |  |  |  | 1 |  |  |  | 1 |
| 14 |  |  |  | 1 |  |  |  |  | 1 |
| 15 |  |  |  |  |  | 1 |  |  | 1 |
| 16 |  | 1 |  |  |  |  |  |  | 1 |
| 36 |  | 3 |  |  |  |  |  |  | 3 |
| 86 | 1 |  |  |  |  |  |  |  | 1 |
| 147 | 2 |  |  |  |  |  |  |  | 2 |
| 289 | 2 |  |  |  |  |  |  |  | 2 |
| 437 |  |  |  |  |  | 1 |  |  | 1 |
| Novel | 1 |  |  |  |  |  |  |  | 1 |

## Supplementary Table 3. The *in vitro* activity of aztreonam/nacubactam, cefepime/nacubactam, and comparator agents against isolates with siderophore uptake deficiencies and those isolates with such deficiencies that were cefiderocol-nonsusceptible (EUCAST).

| **Drug** | **All (36)** | | | | | | **FDC-NS (27)** | | | | | |
| --- | --- | --- | --- | --- | --- | --- | --- | --- | --- | --- | --- | --- |
|  | **MIC (mg/L)** | | | **%Sus** | | **%≤4/4 mg/L (ANC)** | **MIC (mg/L)** | | | **%Sus** | | **%≤4/4 mg/L (ANC)** |
|  | **MIC₅₀** | **MIC₉₀** | **Range** | **CLSI** | **EUCAST** | **%≤8/8 mg/L (FNC)** | **MIC₅₀** | **MIC₉₀** | **Range** | **CLSI** | **EUCAST** | **%≤8/8 mg/L (FNC)** |
| ANC | 1 | 2 | 0.12 - 2 | na | na | 100 | 1 | 2 | 0.25 - 2 | na | na | 100 |
| ATM | 32 | >64 | 1 - >64 | 13.9 | 5.6 | na | >64 | >64 | 2 - >64 | 7.4 | 0.0 | na |
| FNC | 2 | 4 | 0.25 - 8 | na | na | 100 | 2 | 4 | 0.25 - 4 | na | na | 100 |
| FEP | >64 | >64 | 0.5 - >64 | 2.8 | 2.8 | na | >64 | >64 | 0.5 - >64 | 3.7 | 3.7 | na |
| AZA | 0.25 | 8 | ≤0.015 - 16 | 88.9 | 88.9 | na | 0.5 | 8 | 0.06 - 16 | 85.2 | 85.2 | na |
| CZA | >64 | >64 | 0.06 - >64 | 27.8 | 27.8 | na | >64 | >64 | 0.5 - >64 | 29.6 | 29.6 | na |
| CAZ | >32 | >32 | 4 - >32 | 2.8 | 0.0 | na | >32 | >32 | 16 - >32 | 0.0 | 0.0 | na |
| FDC | 4 | >32 | 1 - >32 | 50.0 | 25.0 | na | 8 | >32 | 4 - >32 | 33.3 | 0.0 | na |
| IMR | 16 | >32 | 0.25 - >32 | 13.9 | 16.7 | na | 16 | >32 | 0.5 - >32 | 7.4 | 11.1 | na |
| IPM | 16 | >32 | 2 - >32 | 0.0 | 5.6 | na | 16 | >32 | 2 - >32 | 0.0 | 3.7 | na |
| MEV | >16 | >16 | 0.03 - >16 | 19.4 | 22.2 | na | >16 | >16 | 0.06 - >16 | 14.8 | 18.5 | na |
| MEM | >16 | >16 | 0.5 - >16 | 2.8 | 5.6 | na | >16 | >16 | 0.5 - >16 | 3.7 | 3.7 | na |
| TZP | >32 | >32 | 32 - >32 | 0.0 | 0.0 | na | >32 | >32 | >32 - >32 | 0.0 | 0.0 | na |
| CST | 0.25 | >16 | ≤0.12 - >16 | 0.0 | 75 | na | 0.25 | >16 | ≤0.12 - >16 | 0.0 | 77.8 | na |

Siderophore uptake deficiencies were premature stop codons in *cirA* (n=28) and *fiu* (n=8)

Abbreviations: %Sus, percent susceptible; ANC, aztreonam/nacubactam; ATM, aztreonam; FNC, cefepime/nacubactam; FEP, cefepime; AZA, aztreonam/avibactam; CZA, ceftazidime/avibactam; CAZ, ceftazidime; FDC, cefiderocol; IMR, imipenem/relebactam; IPM, imipenem; MEV, meropenem/vaborbactam; MEM, meropenem.

Supplementary Table 4. The *in vitro* activity of aztreonam/nacubactam, cefepime/nacubactam, and comparator agents against carbapenemase-positive isolates with mutations in the genes encoding OmpC and/or OmpF and their homologs.

| **Drug** | **Porin mutation (number of isolates)** | | | | | | | | | | | | | | | | | | | |
| --- | --- | --- | --- | --- | --- | --- | --- | --- | --- | --- | --- | --- | --- | --- | --- | --- | --- | --- | --- | --- |
|  | **No mutation (181)** | | | | | **OmpC+F-mut (261)** | | | | | **OmpC-mut (76)** | | | | | **OmpF-mut (112)** | | | | |
|  | **MIC₅₀** | **MIC₉₀** | **%Sus** | | **%≤4/4 mg/L (ANC)** | **MIC₅₀** | **MIC₉₀** | **%Sus** | | **%≤4/4 mg/L (ANC)** | **MIC₅₀** | **MIC₉₀** | **%Sus** | | **%≤4/4 mg/L (ANC)** | **MIC₅₀** | **MIC₉₀** | **%Sus** | | **%≤4/4 mg/L (ANC)** |
|  |  |  | **CLSI** | **EUCAST** | **%≤8/8 mg/L (FNC)** |  |  | **CLSI** | **EUCAST** | **%≤8/8 mg/L (FNC)** |  |  | **CLSI** | **EUCAST** | **%≤8/8 mg/L (FNC)** |  |  | **CLSI** | **EUCAST** | **%≤8/8 mg/L (FNC)** |
| ANC | 1 | 2 | na | na | 98.9 | 2 | 2 | na | na | 99.2 | 1 | 2 | na | na | 100 | 1 | 2 | na | na | 99.1 |
| ATM | >64 | >64 | 13.8 | 9.4 | na | >64 | >64 | 2.7 | 2.7 | na | >64 | >64 | 7.9 | 2.6 | na | >64 | >64 | 11.6 | 7.1 | na |
| FNC | 2 | 4 | na | na | 96.7 | 2 | 4 | na | na | 97.3 | 2 | 8 | na | na | 92.1 | 2 | 4 | na | na | 98.2 |
| FEP | >64 | >64 | 3.3 | 2.2 | na | >64 | >64 | 0.8 | 0.4 | na | >64 | >64 | 0.0 | 0.0 | na | >64 | >64 | 4.5 | 3.6 | na |
| AZA | 0.12 | 0.5 | na | 98.9 | na | 0.25 | 0.5 | na | 98.5 | na | 0.25 | 8 | na | 85.5 | na | 0.25 | 0.5 | na | 99.1 | na |
| CZA | >64 | >64 | 32.6 | 32.6 | na | 2 | >64 | 60.9 | 60.9 | na | >64 | >64 | 43.4 | 43.4 | na | >64 | >64 | 38.4 | 38.4 | na |
| CAZ | >32 | >32 | 2.8 | 1.1 | na | >32 | >32 | 2.3 | 1.9 | na | >32 | >32 | 3.9 | 0.0 | na | >32 | >32 | 3.6 | 1.8 | na |
| FDC | 2 | 8 | 87.8 | 64.6 | na | 1 | 4 | 97.3 | 82.8 | na | 2 | >32 | 80.3 | 51.3 | na | 2 | 4 | 93.8 | 75.0 | na |
| IMR | 8 | 32 | 26.0 | 30.9 | na | 4 | >32 | 30.7 | 42.1 | na | 16 | >32 | 7.9 | 15.8 | na | 8 | 32 | 32.1 | 37.5 | na |
| IPM | 16 | >32 | 3.3 | 9.4 | na | >32 | >32 | 0.8 | 2.7 | na | 16 | >32 | 1.3 | 2.6 | na | 16 | >32 | 2.7 | 8.0 | na |
| MEV | 8 | >16 | 44.2 | 53.6 | na | >16 | >16 | 27.2 | 32.2 | na | >16 | >16 | 10.5 | 13.2 | na | 16 | >16 | 40.2 | 47.3 | na |
| MEM | 16 | >16 | 7.2 | 16.0 | na | >16 | >16 | 0.0 | 0.8 | na | >16 | >16 | 2.6 | 2.6 | na | >16 | >16 | 0.9 | 4.5 | na |
| TZP | >32 | >32 | 0.0 | 0.0 | na | >32 | >32 | 0.4 | 0.4 | na | >32 | >32 | 1.3 | 1.3 | na | >32 | >32 | 0.9 | 0.9 | na |
| CST | 0.25 | >16 | 0.0 | 79.0 | na | 0.25 | 16 | 0.0 | 74.3 | na | 0.25 | 16 | 0.0 | 81.6 | na | 0.25 | 8 | 0.0 | 89.3 | na |

Abbreviations: %Sus, percent susceptible; ANC, Aztreonam/nacubactam; ATM, Aztreonam; FNC, Cefepime/nacubactam; FEP, Cefepime; AZA, Aztreonam/avibactam; CZA, Ceftazidime/avibactam; CAZ, Ceftazidime; FDC, Cefiderocol; IMR, Imipenem/relebactam; IPM, Imipenem; MEV, Meropenem/vaborbactam; MEM, Meropenem.

OmpC includes homologs OmpK36 and Omp36. OmpF includes homologs OmpK35 and Omp35.

Mutations were defined as 1) previously described mutations that occlude the eyelet of the porin: *K. pneumoniae* OmpK36 ~133 insertion of "G", "D", "GD", or "DT"; *E. coli* OmpC G137D; 2) mutations leading to a premature stop codon (frameshift or nonsense); or 3) identification of an insertion sequence in the coding region of the gene.

## Supplementary Table 5. The *in vitro* activity of aztreonam/nacubactam, cefepime/nacubactam, and comparator agents against six isolates that produced variants of KPC and were resistant to ceftazidime-avibactam.

| **Year** | **Organism** | **Country** | **Inf. Source** | **ANC** | **ATM** | **AZA** | **CAZ** | **CZA** | **FDC** | **FEP** | **FNC** | **IMR** | **MEM** | **MEV** | **MLST** | **Beta-lactamase summary** | **Chromosomal resistance** |
| --- | --- | --- | --- | --- | --- | --- | --- | --- | --- | --- | --- | --- | --- | --- | --- | --- | --- |
| 2023 | *Providencia rettgeri* | United States | LRT | 0.12 | 1 | 0.03 | 16 | 16 | 2 | 16 | 8 | 0.5 | >16 | 0.25 | NA | CARB-2; TEM-1; TEM-Trunc; KPC-2 | ΔcirA |
| 2022 | *Klebsiella pneumoniae* | Italy | IA | 1 | 16 | 1 | >32 | >64 | 8 | 32 | 1 | 0.12 | 2 | 0.5 | 101 | SHV-1; KPC-31 | ompC-occ, ΔompF; ΔacrR |
| 2022 | *Klebsiella pneumoniae* | Italy | UT | 1 | >64 | 0.25 | >32 | 16 | 2 | 64 | 1 | 0.12 | >16 | 0.5 | 101 | SHV-1; KPC-49 | ompC-occ, ΔompF; ΔacrR |
| 2022 | *Klebsiella pneumoniae* | Italy | Blood | 1 | >64 | 0.25 | >32 | 16 | 2 | 64 | 1 | 0.12 | >16 | 0.5 | 101 | SHV-1; KPC-49 | ompC-occ, ΔompF; ΔacrR |
| 2021 | *Klebsiella pneumoniae* | Croatia | Blood | 2 | >64 | 2 | >32 | >64 | 1 | >64 | 2 | 2 | >16 | 8 | 101 | SHV-1; TEM-1; OXA-9-Trunc; KPC-2; KPC-97 | ompC-occ, ΔompF |
| 2021 | *Klebsiella pneumoniae* | Croatia | UT | 2 | >64 | 4 | >32 | >64 | 2 | >64 | 2 | 1 | >16 | 8 | 101 | SHV-1; TEM-1; OXA-9-Trunc; KPC-2; KPC-New variant | ompC-occ, ΔompF |

Abbreviations: ANC, aztreonam/nacubactam; ATM, aztreonam; AZA, aztreonam-avibactam; CAZ, ceftazidime; CZA, ceftazidime-avibactam; FDC, cefiderocol; FEP, cefepime; FNC, cefepime/nacubactam; IMR, imipenem-relebactam; MEM, meropenem; MEV, meropenem-vaborbactam; MLST, multilocus sequence type; LRT, lower respiratory tract; IA, intra-abdominal; UT, urinary tract; Trunc, truncated, i.e. a premature stop codon was detected in the coding sequence; occ, occluded, a previously-described mutation was found that occludes the eyelet of the porin (e.g. insertion of Gly-Asp at 133); Δ, loss through premature stop codon or insertion sequence.

## Supplementary Figure 1. Cumulative percent of MBL-positive isolates inhibited by increasing concentrations of agents, by variant

MBL-positive (330)

Percent inhibited

| Agent concentration (mg/L) | ANC | FNC | IMR | CZA | MEV | AZA | FDC |
| --- | --- | --- | --- | --- | --- | --- | --- |
| ≤0.03 | 0.3 | 0.3 |  |  | 0.3 | 7.6 | 0.3 |
| 0.06 | 2.4 | 0.3 | 0.3 | 0.3 | 0.3 | 21.5 | 0.6 |
| 0.12 | 5.2 | 0.3 | 0.3 | 0.3 | 0.3 | 48.5 | 2.4 |
| 0.25 | 12.4 | 0.3 | 0.3 | 0.6 | 0.9 | 75.5 | 3.9 |
| 0.5 | 27.3 | 0.3 | 1.2 | 0.6 | 1.8 | 87.3 | 12.1 |
| 1 | 73 | 19.4 | 1.8 | 1.2 | 3.6 | 90.3 | 26.1 |
| 2 | 96.4 | 71.5 | 5.2 | 1.5 | 6.7 | 92.1 | 57 |
| 4 | 98.5 | 87 | 10.6 | 1.8 | 12.1 | 95.2 | 86.1 |
| 8 | 99.4 | 93.6 | 24.5 | 1.8 | 18.8 | 97.9 | 93 |
| 16 | 99.4 | 95.8 | 47.3 | 2.1 | 30.3 | 99.1 | 96.1 |
| 32 | 99.4 | 97.6 | 67.9 | 3 |  | 99.1 | 96.7 |
| 64 | 99.4 | 99.4 |  | 5.5 |  | 99.7 |  |

NDM-1 (174)

|  | ANC | FNC | IMR | CZA | MEM/VAB8 | AZA | FDC |
| --- | --- | --- | --- | --- | --- | --- | --- |
| ≤0.03 | 0.6 | 0 |  |  | 0 | 5.7 | 0 |
| 0.06 | 2.3 | 0 | 0 | 0.6 | 0 | 20.7 | 0 |
| 0.12 | 4.6 | 0 | 0 | 0.6 | 0 | 60.3 | 2.3 |
| 0.25 | 14.4 | 0 | 0.6 | 0.6 | 0 | 90.2 | 4 |
| 0.5 | 28.7 | 0 | 0.6 | 0.6 | 0 | 97.1 | 10.3 |
| 1 | 79.3 | 9.2 | 0.6 | 0.6 | 0.6 | 97.7 | 24.7 |
| 2 | 97.1 | 65.5 | 4 | 0.6 | 4.6 | 98.3 | 55.7 |
| 4 | 98.9 | 83.9 | 6.9 | 1.1 | 9.8 | 98.9 | 89.7 |
| 8 | 100 | 92.5 | 20.7 | 1.1 | 18.4 | 99.4 | 96 |
| 16 | 100 | 94.3 | 43.7 | 1.1 | 32.2 | 99.4 | 100 |
| 32 | 100 | 96.6 | 70.1 | 1.1 |  | 99.4 | 100 |
| 64 | 100 | 98.9 |  | 1.7 |  | 100 |  |

NDM-4 (12)

|  | ANC | FNC | IMR | CZA | MEM/VAB8 | AZA | FDC |
| --- | --- | --- | --- | --- | --- | --- | --- |
| ≤0.03 | 0.3 | 0.3 |  |  | 0.3 | 16.7 | 0.3 |
| 0.06 | 0.3 | 0.3 | 0.3 | 0.3 | 0.3 | 50 | 0.3 |
| 0.12 | 0.3 | 0.3 | 0.3 | 0.3 | 0.3 | 58.3 | 8.3 |
| 0.25 | 25 | 0.3 | 0.3 | 0.3 | 0.3 | 75 | 8.3 |
| 0.5 | 41.7 | 0.3 | 0.3 | 0.3 | 0.3 | 83.3 | 8.3 |
| 1 | 83.3 | 25 | 0.3 | 8.3 | 0.3 | 100 | 16.7 |
| 2 | 100 | 91.7 | 8.3 | 16.7 | 0.3 | 100 | 50 |
| 4 | 100 | 100 | 16.7 | 16.7 | 0.3 | 100 | 91.7 |
| 8 | 100 | 100 | 25 | 16.7 | 0.3 | 100 | 91.7 |
| 16 | 100 | 100 | 41.7 | 16.7 | 16.7 | 100 | 100 |
| 32 | 100 | 100 | 83.3 | 16.7 |  | 100 | 100 |
| 64 | 100 | 100 |  | 16.7 |  | 100 |  |

NDM-5 (115)

|  | ANC | FNC | IMR | CZA | MEM/VAB8 | AZA | FDC |
| --- | --- | --- | --- | --- | --- | --- | --- |
| ≤0.03 | 0.3 | 0.3 |  |  | 0.3 | 7.8 | 0.9 |
| 0.06 | 3.5 | 0.3 | 0.3 | 0.3 | 0.3 | 14.8 | 0.9 |
| 0.12 | 4.3 | 0.3 | 0.3 | 0.3 | 0.3 | 27.8 | 0.9 |
| 0.25 | 6.1 | 0.3 | 0.3 | 0.9 | 0.3 | 52.2 | 0.9 |
| 0.5 | 16.5 | 0.3 | 0.9 | 0.9 | 0.3 | 70.4 | 9.6 |
| 1 | 60 | 33.9 | 1.7 | 1.7 | 1.7 | 76.5 | 24.3 |
| 2 | 94.8 | 76.5 | 3.5 | 1.7 | 1.7 | 80.9 | 54.8 |
| 4 | 98.3 | 89.6 | 9.6 | 1.7 | 6.1 | 88.7 | 78.3 |
| 8 | 99.1 | 95.7 | 21.7 | 1.7 | 9.6 | 95.7 | 87.8 |
| 16 | 99.1 | 97.4 | 46.1 | 1.7 | 18.3 | 99.1 | 89.6 |
| 32 | 99.1 | 98.3 | 57.4 | 2.6 |  | 99.1 | 90.4 |
| 64 | 99.1 | 100 |  | 6.1 |  | 100 |  |

VIM-1 (14)

|  | ANC | FNC | IMR | CZA | MEM/VAB8 | AZA | FDC |
| --- | --- | --- | --- | --- | --- | --- | --- |
| ≤0.03 | 0 | 0 |  | 0 | 0 | 14.3 | 0 |
| 0.06 | 0 | 0 | 0 | 0 | 0 | 42.9 | 0 |
| 0.12 | 21.4 | 0 | 0 | 0 | 0 | 64.3 | 0 |
| 0.25 | 35.7 | 0 | 0 | 0 | 0 | 85.7 | 7.1 |
| 0.5 | 64.3 | 0 | 0 | 0 | 7.1 | 100 | 28.6 |
| 1 | 78.6 | 7.1 | 0 | 0 | 21.4 | 100 | 50 |
| 2 | 100 | 78.6 | 0 | 0 | 35.7 | 100 | 85.7 |
| 4 | 100 | 85.7 | 28.6 | 0 | 50 | 100 | 92.9 |
| 8 | 100 | 85.7 | 64.3 | 0 | 71.4 | 100 | 92.9 |
| 16 | 100 | 100 | 85.7 | 7.1 | 85.7 | 100 | 92.9 |
| 32 | 100 | 100 | 100 | 7.1 |  | 100 | 100 |
| 64 | 100 | 100 |  | 14.3 |  | 100 |  |

Supplementary Figure 2. Cumulative percent of serine-carbapenemase-positive isolates inhibited by increasing concentrations of agents, by variant

KPC-2 (91)

|  | ANC | FNC | IMR | CZA | MEM/VAB8 | AZA | FDC |
| --- | --- | --- | --- | --- | --- | --- | --- |
| ≤0.03 | 0 | 0 |  | 0 | 25.6 | 3.3 | 2.2 |
| 0.06 | 0 | 0 | 3.3 | 1.1 | 35.6 | 5.6 | 4.4 |
| 0.12 | 1.1 | 0 | 28.9 | 1.1 | 40 | 24.4 | 11.1 |
| 0.25 | 2.2 | 0 | 63.3 | 7.8 | 52.2 | 64.4 | 22.2 |
| 0.5 | 2.2 | 22.2 | 87.8 | 25.6 | 64.4 | 88.9 | 38.9 |
| 1 | 41.1 | 56.7 | 93.3 | 70 | 75.6 | 96.7 | 72.2 |
| 2 | 96.7 | 97.8 | 98.9 | 92.2 | 84.4 | 97.8 | 94.4 |
| 4 | 100 | 98.9 | 98.9 | 95.6 | 87.8 | 100 | 98.9 |
| 8 | 100 | 100 | 100 | 96.7 | 97.8 | 100 | 98.9 |
| 16 | 100 | 100 | 100 | 97.8 | 97.8 | 100 | 100 |
| 32 | 100 | 100 | 100 | 97.8 |  | 100 | 100 |
| 64 | 100 | 100 |  | 97.8 |  | 100 |  |

KPC-3 (62)

|  | ANC | FNC | IMR | CZA | MEM/VAB8 | AZA | FDC |
| --- | --- | --- | --- | --- | --- | --- | --- |
| ≤0.03 | 0 | 0 |  | 1.6 | 26.2 | 4.9 | 0 |
| 0.06 | 0 | 0 | 0 | 1.6 | 31.1 | 8.2 | 0 |
| 0.12 | 0 | 1.6 | 29.5 | 1.6 | 31.1 | 21.3 | 23 |
| 0.25 | 1.6 | 1.6 | 75.4 | 1.6 | 37.7 | 73.8 | 37.7 |
| 0.5 | 1.6 | 24.6 | 93.4 | 13.1 | 67.2 | 100 | 49.2 |
| 1 | 49.2 | 72.1 | 95.1 | 42.6 | 91.8 | 100 | 68.9 |
| 2 | 96.7 | 98.4 | 100 | 88.5 | 96.7 | 100 | 98.4 |
| 4 | 100 | 100 | 100 | 100 | 100 | 100 | 100 |
| 8 | 100 | 100 | 100 | 100 | 100 | 100 | 100 |
| 16 | 100 | 100 | 100 | 100 | 100 | 100 | 100 |
| 32 | 100 | 100 | 100 | 100 |  | 100 | 100 |
| 64 | 100 | 100 |  | 100 |  | 100 |  |

OXA-48 (73)

|  | ANC | FNC | IMR | CZA | MEM/VAB8 | AZA | FDC |
| --- | --- | --- | --- | --- | --- | --- | --- |
| ≤0.03 | 0 | 0 |  | 0 | 0 | 1.4 | 2.7 |
| 0.06 | 0 | 0 | 0 | 0 | 0 | 1.4 | 5.5 |
| 0.12 | 0 | 0 | 0 | 0 | 0 | 17.8 | 12.3 |
| 0.25 | 5.5 | 1.4 | 1.4 | 0 | 0 | 72.6 | 15.1 |
| 0.5 | 15.1 | 13.7 | 5.5 | 34.2 | 1.4 | 90.4 | 26 |
| 1 | 47.9 | 56.2 | 9.6 | 75.3 | 5.5 | 98.6 | 50.7 |
| 2 | 95.9 | 90.4 | 24.7 | 90.4 | 12.3 | 98.6 | 78.1 |
| 4 | 100 | 100 | 56.2 | 98.6 | 16.4 | 98.6 | 95.9 |
| 8 | 100 | 100 | 75.3 | 98.6 | 21.9 | 98.6 | 98.6 |
| 16 | 100 | 100 | 86.3 | 98.6 | 50.7 | 98.6 | 100 |
| 32 | 100 | 100 | 89 | 98.6 |  | 98.6 | 100 |
| 64 | 100 | 100 |  | 98.6 |  | 98.6 |  |

OXA-232 (44)

|  | ANC | FNC | IMR | CZA | MEM/VAB8 | AZA | FDC |
| --- | --- | --- | --- | --- | --- | --- | --- |
| ≤0.03 | 0 | 0 |  | 0 | 0 | 2.3 | 2.3 |
| 0.06 | 0 | 0 | 0 | 2.3 | 0 | 4.5 | 2.3 |
| 0.12 | 0 | 0 | 0 | 2.3 | 0 | 11.4 | 4.5 |
| 0.25 | 0 | 0 | 2.3 | 2.3 | 0 | 63.6 | 11.4 |
| 0.5 | 4.5 | 2.3 | 4.5 | 25 | 0 | 95.5 | 22.7 |
| 1 | 54.5 | 54.5 | 15.9 | 77.3 | 0 | 100 | 61.4 |
| 2 | 100 | 100 | 45.5 | 97.7 | 4.5 | 100 | 90.9 |
| 4 | 100 | 100 | 84.1 | 97.7 | 9.1 | 100 | 100 |
| 8 | 100 | 100 | 93.2 | 97.7 | 11.4 | 100 | 100 |
| 16 | 100 | 100 | 100 | 97.7 | 31.8 | 100 | 100 |
| 32 | 100 | 100 | 100 | 97.7 |  | 100 | 100 |
| 64 | 100 | 100 |  | 97.7 |  | 100 |  |
